# Lactobacilli Are Prominent Members of the Microbiota Involved in the Ruminal Digestion of Barley and Corn

**DOI:** 10.3389/fmicb.2018.00718

**Published:** 2018-04-10

**Authors:** Hee E. Yang, Claiton A. Zotti, John J. McKinnon, Tim A. McAllister

**Affiliations:** ^1^Lethbridge Research and Development Centre, Agriculture and Agri-Food Canada, Lethbridge, AB, Canada; ^2^Department of Animal and Poultry Science, University of Saskatchewan, Saskatoon, SK, Canada; ^3^Department of Animal Science, Universidade do Oeste de Santa Catarina, Xanxerê, Xanxerê, Brazil

**Keywords:** barley, corn, rumen, microbiome, starch digestion, cattle, biofilm

## Abstract

The chemical composition of barley grain can vary among barley varieties (Fibar, Xena, McGwire, and Hilose) and result in different digestion efficiencies in the rumen. It is not known if compositional differences in barley can affect the microbiota involved in the ruminal digestion of barley. The objective of this study was to characterize the *in situ* rumen degradability and microbiota of four barley grain varieties and to compare these to corn. Three ruminally cannulated heifers were fed a low (60% barley silage, 37% barley grain, and 3% supplement) or high grain (37% barley silage, 60% barley grain, and 3% supplement) diet. One set of bags was used to estimate dry matter (DM), starch and crude protein (CP) degradability. A second set was used to extract DNA from the adherent microbiota and visualize grain after incubation using scanning electron microscopy (SEM). DNA was subjected to amplicon 16S rRNA gene sequencing followed by analysis using QIIME. In the low grain diet, McGwire had the highest effective degradability (ED) of DM (*P* < 0.01). The ED of starch was highest (*P* < 0.01) for Fibar, McGwire, and Xena, but the ED of CP was not affected by variety. For the high grain diet, Xena and McGwire had the highest ED of DM (*P* < 0.01). The ED of starch was highest (*P* < 0.01) for Xena and Fibar. The ED of protein was highest (*P* < 0.01) for Xena and McGwire. Although the microbiota did not differ among barley varieties, they did differ from corn and with incubation time. Lactobacilli were dominant members of the mature biofilms associated with corn and barley and were accompanied by a notable increase in the lactic acid utilizing genera, *Megasphaera*. As none of the cattle exhibited subclinical or clinical acidosis during the study, our results suggest that lactobacilli play a more prominent role in routine starch digestion than presently surmised.

## Introduction

Barley is ranked as the fourth major cereal crop in the world consumed by humans and livestock (Nikkhah, 2012) and is the primary grain fed to ruminants in Western Canada (Nikkhah, 2012). On average, barley grain is composed of ≈ 60% starch, 20% fiber and 12% protein on a DM basis (Åman and Newman, 1986; Oscarsson et al., 1996). However, the chemical composition, nutritive value, and bioavailability of starch can vary among barley varieties due to genetic and environmental factors (Nilan and Ullrich, 1993). Barley grain can be classified as two or six row, hulled or hulless, and on the basis of starch type as normal, waxy (high amylopectin up to 100%), or high-amylose (amylose up to 70%) (Kong et al., 1995; Narasimhalu et al., 1995). Variations in the chemical composition and structure of barley grain can affect its ruminal digestibility (Cleary et al., 2011).

Ruminal digestion of barley is dependent on the ability of rumen microbes to colonize and form biofilms on the surface of the grain (McAllister et al., 1994; Beauchemin et al., 2001). Formation of biofilms can be promoted by processing barley grain which enhances microbial attachment and the colonization of starch granules (McAllister et al., 1994). However, even when processed using the same techniques, ruminal digestion can differ among barley varieties. Little is known about how differences in barley grain composition affect the types of rumen bacteria that form biofilms on the surface of barley grain. Previous studies of the microbiota involved in the ruminal digestion of barley grain have primarily relied on culture-based methods and were therefore limited to only those bacteria that could be grown in the laboratory. Recent developments in molecular biology has led to a proliferation in the use of next generation sequencing (NGS) to characterize the ecology of microbial populations in a variety of ecological habitats, including the rumen (Petri et al., 2013). NGS is a culture-free method that enables analysis of the entire bacterial population based on sequencing of the16S rRNA gene. It enables the characterization of the phylogeny and taxonomy of bacteria including those that are associated with complex biofilms. The objectives of this study were (i) to estimate the temporal *in situ* rumen degradability of four barley grain varieties (Fibar, Xena, McGwire, and Hilose) and (ii) evaluate the temporal formation of bacterial biofilms involved in the digestion of these barley grain varieties. We hypothesized that the population structure of rumen microbial biofilms would differ among barley varieties as a result of differences in their chemical composition. Corn was included in the study as a positive control as it is well known that the chemical composition of corn differs dramatically from barley (Peter and Herbert, 2013) and grain colonization was investigated using heifers fed both low and high grain diets.

## Materials and Methods

### Animal and Diets

Three rumen-cannulated heifers (BW: 308 kg ± 32 SD) were individually housed for the duration of the experiment with free access to clean drinking water. The heifers were fed *ad libitum* twice daily at 08:00 and 16:00 h. The study was reviewed and approved by the Lethbridge Research Centre Animal Care Committee and conducted according to the guidelines of the Canadian Council on Animal Care [CCAC] (2009). In the first experiment, heifers were adapted to a low grain diet (DM basis) consisting of 60% barley silage, 37% steam rolled barley and 3% of a standard feedlot supplement for a period of 21 days (**Table 1**). *In situ* incubations were conducted and then heifers were adapted to a high grain diet over 14 days. In the second experiment, the diet consisted of 37% barley silage, 60% steam rolled barley and 3% supplement (**Table 1**). Heifers were fed the second diet for an additional 14 days prior to *in situ* incubations.

**Table 1:** Diet composition.

Composition	Low concentrate	High concentrate
**Ingredients (% of dry matter)**		
Barley silage	60	37
Barley grain	37	60
Supplement ^a^	3	3
**Composition (% of dry matter**		
Crude protein	12.0	12.1
Acid detergent fiber	24.9	11.8
Neutral detergent fiber	36.2	19.7
Starch	16.7	35.2

*^*a*^The supplement contained canola meal (10%), urea (2%), calcium carb (25%), sodium chloride (3%), ground barley grain (56.5%), molasses (2.5%) and feed lot premix (1%). The premix contained calcium carbonate (34.8%), zinc sulfate (28.4%), manganous sulfate (14.6%), copper sulfate (10.3%), ethylene diaminediiodic acid (0.2%; as an 80% preparation), selenium (5%), cobalt sulfate (0.1%), vitamin A (1,000,000 IU g^-*1*^; 1.7%), vitamin D (500,000 IU g^-*1*^; 0.2%) and vitamin E (4.7%).*

### Incubation of Barley Varieties and Corn in the Rumen

Prior to placement in nylon bags, barley varieties (Fibar, Xena, McGwire, and Hilose) and corn were ground using a Wiley mill (Arthur H. Thomas Co.) fitted with a 6 mm screen. A 6 mm screen was selected to ensure that both compositional and structural traits of the grain influenced biofilm formation on the grain surface. It also facilitated the collection of grain particles for examination using scanning electron microscopy (SEM). *In situ* dry matter (DM), starch and crude protein (CP) disappearance were estimated using three heifers. Samples were hot weighted (3 g) into 50 μm pore size (#R510) Dacron nylon bags (5 cm × 10 cm; Ankon, Fairport, NY, United States), that were placed in a larger mesh bag and removed from the rumen after 0, 2, 4, 12, 24, and 48 h. The same nylon bags containing grains (5 g) for DNA extraction and SEM were incubated in two of the heifers and removed from the rumen after 2, 4, and 12 h. Bags used to estimate *in situ* disappearance were retrieved from the rumen and thoroughly rinsed with cold tap water until the water was clear. Bags were then washed in a washing machine for 2 min without the spin cycle, prior to being dried at 55°C for 48 h. For DNA extraction, samples were rinsed with phosphate buffer three times to remove non-adherent bacteria and contents were flash-frozen in liquid nitrogen. For SEM, two or three random particles from the bags were placed into a prefix solution containing 0.5% glutaraldehyde, 0.15% ruthenium red in 0.2M phosphate buffer saline at a pH of 7.2. Zero hour bags were not placed in the rumen, but were washed in the same manner as the other bags.

### DNA Extraction of Bacterial Biofilms

Samples for DNA extraction (*n* = 3) were freeze-dried and 2–4 g were ground for 5 min at a frequency of 30 cycles/s using a Qiagen TissueLyser II (Qiagen, Toronto, ON, United States). DNA was extracted using a Qiagen QIAamp DNA stool mini kit with minor modifications. Briefly, 300 mg of ground substrate were placed into a 2 ml micro centrifuge tube along with 1.4 ml of ASL buffer (cell lysis) and the mixture was vortexed for 1 min. Glass beads (200 mg with 0.5 mm diameter and 300 mg with 1.0 mm diameter) were added to each tube and the samples were processed in a bead-beating homogenizer (B. Braun, Melsungen AG, Germany) for 3 min at maximum amplitude. The tubes were then centrifuged (13,000 × *g*, 5 min) and subsequently extracted following the QIAamp DNA stool mini kit instructions. After extraction, DNA was quantified using a NanoDrop 3300 (Thermo, Waltham, MA, United States).

### Pyrosequencing of the 16S rRNA Gene

Extracted DNA was analyzed using bacterial tag-encoded FLX 16s rRNA amplicon pyrosequencings (bTEFAP). The 16S rRNA gene universal bacterial primers 27F-519R (27F 5’-AGRGTTTGATCMTGGCTCAG, 519R 5’-GTNTTACNGCGGCKGCTG), were used to amplify the hypervariable regions V1 to V3 of the 16S rRNA gene as described by Dowd et al. (2008). All DNA samples were adjusted to100 ng/μl and an 1 μl aliquot (100 ng) of each sample of extracted DNA was used in a 50 μl PCR reaction. Reactions were performed using a HotStarTaq plus Master Mix Kit (Qiagen, Valencia, CA, United States) with the following conditions: 94°C for 3 min, followed by 28 cycles of 94°C for 30 s; 53°C for 40 s; and 72°C for 1 min; followed by a final elongation step at 72°C for 5 min. Following PCR, all amplicon products from different samples were mixed in equal concentrations and purified using Agencourt Ampure beads (Agencourt Bioscience Corporation, Beverly, MA, United States). Prepared libraries were sequenced utilizing Roche 454 FLX titanium (454 Life Sciences, a Roche company, Branford, CT, United States) technology with reagents and execution of the procedure as per manufacturer’s guidelines. Sequence data was submitted to short read database of NCBI and awarded accession SRP132480.

### Sequencing Analysis by QIIME 1.9.1

16S rRNA gene sequences were processed and analyzed with the QIIME software package v. 1.9.1 (Caporaso et al., 2010). All samples were demultiplexed and sequences were quality filtered for, low quality or ambiguous reads and homopolymers and sequences with an average Phred score of <25 were removed. Chimeric sequences were removed using the UCHIME algorithm implemented in USEARCH (version 6.1544). The remaining high quality 16S rRNA gene sequences were clustered into OTUs (operational taxonomic units) at 97% similarity using the *de novo* reference OTU picking method and USEARCH (version 6.1544). Taxonomy was assigned using the UCLUST consensus taxonomy assigner (Edgar, 2010). PyNAST (Caporaso et al., 2010) was used to align the representative sequences for each OTU and a phylogenetic tree was created using FastTree (Price et al., 2010).

### Chemical Analysis of Barley Grain Varieties and Corn

Briefly, lipids were determined using the Goldfisch extraction apparatus (Labconco Corporation, Kansas, MO, United States) with petroleum ether as the solvent (AOAC, 2010). Total nitrogen (TN) was measured by combustion analysis using a Leco Carbon/Nitrogen analyzer (TruSpec CN, Leco Corporation, St. Joseph, MI, United States), with CP calculated as TN × 6.2. Soluble dietary fiber (SDF) and insoluble dietary fiber (IDF) were quantified using the enzymatic gravimetric procedures of AOAC Methods 993.19 and 991.42, respectively (AOAC, 1997) using Megazyme’s total dietary fiber assay kit (Megazyme International Ireland Ltd., Wicklow, Ireland). Total dietary fiber (TDF) was calculated as SDF plus IDF. Contents of starch and β-glucan were estimated according to the total starch (AA/AMG) and the mixed-linkage β-glucan assays, respectively (Megazyme International Ltd., Bray, Ireland). Amylose content of starch was determined using the iodine blue method as defined by the Corn Refiners Association (1997). Starch content of the grain residues remaining after ruminal incubation was determined by hydrolyzing starch to glucose polymers using amyloglucosidase (Megazyme International Ltd.) plus 1,4-*α*-d-glucan glucano-hydrolase (Brenntag Canada Inc., Toronto, ON, Canada) as described by Herrera-Saldana et al. (1990). For measurement of starch, samples were ground using a ball mill (Mixer Mill MM 2000, Retsch, Germany) to a fine powder. Samples (0.1 g) were weighed into 50-ml test tubes and 25 ml of 0.1 N Na-acetate buffer (pH 5.0, Ca^2+^ 70 ppm) was added. Amylase (200 μl; Termamyl, Novo Nordisk, Bagsvaerd, Denmark) was added and tubes were vortexed immediately and subsequently at 15-min intervals. For starch determination, tubes were incubated in a water bath (98°C) for 1 h to gelatinize and hydrolyze starch. Activated carbon (approximately 0.04 g; Darco_G-60 Activated Carbon, Fisher Scientific Co., No D-127) was added to the tubes, which were then placed for 15 min in a 60°C waterbath and amyloglucosidase (500 μL, Boehringer Mannheim, Laval, QC, Canada, no. 208-469) was added. Samples were centrifuged (695 × *g* for 10 min) and 500 μl of supernatant was diluted with 9.5 ml of distilled water, and 50 μl was transferred to a micro plate. Glucose trinder reagent was added (150 μl, Sigma Chemical Co. No. 315-100), and released glucose was determined colorimetrically at 490 nm using a plate reader.

### Scanning Electron Microscopy (SEM)

Residues of barley grain and corn were fixed in a pre-fix solution (0.5% glutaraldehyde and 0.15% ruthenium red in 0.2M phosphate buffer, pH 7.2). After 2 h, feed particles were removed from the pre-fix solution, and a fix solution of 5% glutaraldehyde and 0.05% ruthenium red in 0.2M phosphate buffer (pH 7.2) was added so as to cover the sample. The fix solution was removed after 2 h and a wash solution (0.2M phosphate buffer and 0.05% ruthenium red, pH 7.2) was added and incubated for 20 min. The wash step was repeated two more times, the final wash solution was removed and a post-fix solution was added (2% OsO_4_ and 0.05% ruthenium red in 0.2M phosphate buffer, pH 7.2). The sample was gently shaken for 2 h and washed five times with wash solution at 10 min intervals. Samples were then dehydrated using a graduate ethanol series containing 10%, 20%, 30% 50%, 70%, 95%, and 100%, ethanol with samples allowed to stand for 15 min at each increment. The specimens were mounted on aluminum stubs with silver paste, coated with gold and observed using a Hitachi S-570 scanning electron microscope (Hitachi High Technologies, Tokyo, Japan) at an accelerating voltage of 19 kV and were photographed with images digitally captured using Quartz PCI software (Quartz Imaging Corporation, Vancouver, BC, Canada).

### *In Situ* Degradability of Four Barley Varieties and Corn

*In situ* DM disappearance of each grain type was determined gravimetrically as the difference between initial and remaining weight of DM after ruminal incubation at each time point. Kinetic parameters of DM, starch, and protein were calculated using the equation (McDonald, 1981):

$$\text{D}\;\text{=}\;\text{a+b}({\text{1-e}}^{\text{-c}(\text{t-lag})})\text{for}\;\text{t}\;>\;\text{lag}$$

Where D = ruminal disappearance at time t, a = the rapidly degradable fraction, b = the slowly degradable fraction, c = the rate at which b was degraded (/h), t = time of incubation (h), and lag = lag time (h). The parameters a, b, c and lag were estimated using NLIN procedure of SAS (SAS 9.3, SAS Institute Inc., Cary, NC, United States). Effective degradability (ED) of DM, starch, and protein were estimated using the equation of Ørskov and McDonald (1979), ED = a + bc/(c+k), where k was the ruminal passage rate of 0.06/h.

All data for each grain type associated with each diet were analyzed as a completely randomized design using PROC MIXED procedure of SAS. For the *in situ* study, the mixed model included the fixed effects of grain type and the random effects of heifer and sample. Replicates within heifer were averaged prior to statistical analysis. Results from the two diet types were analyzed separately as the studies were conducted at different times. Effects of fixed factors were tested using LSMEANS with the PDIFF in SAS and significance was declared at *P* < 0.05. Trends were discussed at 0.05 < *P* < 0.10 unless otherwise stated.

### Rumen Particle-Associated Bacteria Sequencing Analysis

Bacterial diversity within each sample (alpha-diversity) was calculated within QIIME using the Chao1 estimator (Chao, 1984), observed species (observed OTUs), Shannon’s diversity index (Shannon, 1948), PD whole tree [phylogenetic diversity (PD) whole tree] (Faith, 1992), and Goods coverage (Good, 1953) metrics. The bacterial community structure (beta-diversity) within biofilms from all four barley varieties and corn was evaluated using the unweighted and weighted UniFrac distances (Lozupone and Knight, 2005) and visualized as principal coordinate analysis (PCoA) plots using Emperor (Vazquez-Baeza et al., 2013). ANOSIM (analysis of similarities) with 999 permutations was used to compare the unweighted UniFrac distances.

Linear discriminant analysis effect size (LefSe) was used to determine which taxa group differed according to incubation time of corn and barley in heifers fed the low and high grain diet. LefSe includes the Kruskal–Wallis test to identify significantly different (*P* < 0.05) taxa among groups of samples followed by linear discriminant analysis which estimates the effect size of each of these differences (Segata et al., 2011).

## Results

### Chemical Composition of Barley Varieties and Corn

For this study, four barley grain varieties (Fibar, Xena, McGwire, and Hilose) of differing composition and corn were used (**Table 2**). McGwire and Xena were normal starch varieties (approximately 12% amylose and 88% amylopectin); Fibar, was a waxy type with high β-glucan, protein, SDF and, TDF content, but low starch content. Hilose was a high-amylose type with 63% of the starch composed of amylose. Corn, was higher in starch, fat, and IDF than barley, but lower in β-glucan, protein, SDF, and TDF.

**Table 2:** Chemical composition of four different barley grain varieties and corn (values represent mean percentage with standard deviation from triplicate measurements (% of dry matter).

						Dietary fiber		
Grain types	BG^a^	Starch	Amylose	Protein	Fat	SDF^b^	IDF^c^	TDF^d^
Fibar	10.1 ± 0.12	47.8 ± 0.08	0.2 ± 0.01	15.6 ± 0.06	2.6 ± 0.04	7.9 ± 0.17	11.0 ± 0.24	18.9 ± 0.21
McGwire	4.6 ± 0.07	52.5 ± 0.18	11.5 ± 0.88	12.7 ± 0.01	2.0 ± 0.05	5.6 ± 0.38	8.6 ± 0.21	14.2 ± 0.58
Hilose	6.6 ± 0.14	49.0 ± 0.03	18.0 ± 0.25	12.6 ± 0.08	3.0 ± 0.14	7.4 ± 0.31	9.5 ± 0.20	16.9 ± 0.51
Xena	4.1 ± 0.16	56.1 ± 0.15	11.2 ± 0.28	11.4 ± 0.05	1.6 ± 0.05	5.1 ± 0.41	10.4 ± 0.54	15.5 ± 0.15
Corn	0.31 ± 0.00	62.2 ± 0.44	17.1 ± 0.74	7.9 ± 0.03	3.95 ± 0.1	1.14 ± 0.10	12.2 ± 0.25	13.3 ± 0.14

*^*a*^BG, ^*b*^SDF, ^*c*^IDF, and ^*d*^TDF represent β-glucan, soluble dietary fiber, insoluble dietary fiber and total dietary fiber, respectively.*

### Ruminal *in Situ* Degradability of Different Barley Varieties in Heifers Fed Low or High Grain Diets

**FIGURE 1:**
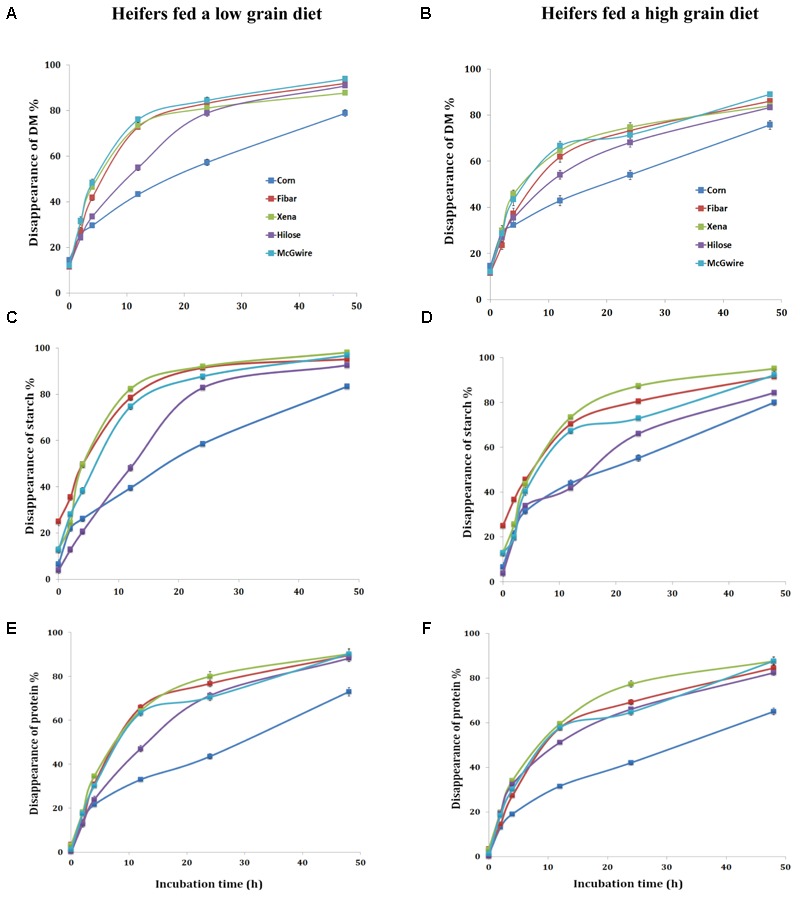
*In situ* percent DM **(A,B)**, starch **(C,D)**, and protein **(E,F)** disappearance at six different ruminal incubation times when heifers were fed a low and high grain diet.

With the low grain diet, the rapid (A) and slow degradable (B) fractions, degradation rate (Kd), the potential degradable fraction (A+B) and ED of DM differed (*P* < 0.01) among grain types (**Table 3**). For DM, the A fraction was the highest (*P* < 0.01) for corn (**Table 3** and **Figure 1A**) and the lowest for Fibar. The fraction B of DM was the highest (*P* < 0.01) for Hilose and lowest for Xena. Degradation rate (Kd) of DM was the fastest (*P* < 0.01) for Xena and slowest for Corn. The potential degradability fraction (A+B) of DM ranked as Hilose > Corn > McGwire > Fibar > Xena. The highest ED values (*P* < 0.01) were observed with McGwire and the lowest with Corn. For starch, the rapidly degradable fraction (A) was highest for Fibar (*P* < 0.01; **Table 3** and **Figure 1C**). Hilose had the highest slowly degradable fraction (B). The Kd of starch was fastest for Xena and Fibar. The ED of starch was highest (*P* < 0.01) for Fibar and Xena, and lowest (*P* < 0.01) for Corn. For protein, the rapidly degradable fraction (A) was highest (*P* < 0.01) for Corn and lowest (*P* < 0.01) for Fibar (**Table 3** and **Figure 1E**). The slowly degradable fraction (B) was highest (*P* < 0.01) for Fibar and lowest for Corn. The Kd of protein was fastest for Fibar and lowest for Corn. However, the ED of protein did not differ among grain types.

**Table 3:** *In situ* degradability estimates of a corn and four barley varieties in heifers fed either a low or high grain diet.

Low grain diet	Corn	Fibar	Hilose	McGwire	Xena	SEM
**Dry matter**						
A (%)	18.16^a^	11.33^c^	13.19^b^	12.47^bc^	12.26^bc^	0.35
B (%)	75.66^bc^	79.60^ab^	81.63^a^	78.22^abc^	73.29^c^	1.34
Kd (/h)	0.04^d^	0.12^b^	0.07^c^	0.14^a^	0.15^a^	0.00
A+B (%)	93.82^a^	91.00^ab^	94.81^a^	90.69^ab^	85.55^b^	1.37
ED (6%/h)	44.99^d^	63.86^b^	55.56^c^	67.49^a^	64.66^b^	0.45
**Starch**						
A (%)	12.50^b^	22.54^a^	7.00^b^	12.04^b^	10.04^b^	1.09
B (%)	85.31^ab^	73.42^b^	91.43^a^	84.87^ab^	87.52^ab^	1.25
Kd (/h)	0.03^d^	0.12^a^	0.07^c^	0.10^b^	0.14^a^	0.00
ED (6%/h)	43.70^c^	70.83^a^	55.48^b^	65.58^a^	70.69^a^	0.68
**Protein**						
A (%)	75.40^a^	52.17^c^	62.79^b^	62.10^b^	66.27^b^	1.09
B (%)	24.60^e^	43.90^a^	36.11^b^	34.41^c^	29.36^d^	0.36
Kd (/h)	0.03^c^	0.14^a^	0.06^ab^	0.10^a^	0.11^a^	0.01
ED (6%/h)	82.76	84.21	80.74	83.90	85.38	1.49

**High grain diet**	**Corn**	**Fibar**	**Hilose**	**McGwire**	**Xena**	**SEM**

**Dry matter**						

A (%)	20.17^a^	11.82^d^	14.74^b^	13.29^c^	13.29^c^	0.35
B (%)	68.82	73.08	70.19	72.24	67.37	1.59
Kd (/h)	0.04^d^	0.10^bc^	0.08^c^	0.13^ab^	0.14^a^	0.01
A+B (%)	88.99^a^	84.90^ab^	84.92^ab^	85.53^ab^	80.66^b^	1.59
ED (6%/h)	44.64^c^	56.61^ab^	52.65^b^	59.05^a^	60.12^a^	1.45
**Starch**						
A (%)	12.22^b^	21.14^a^	10.08^b^	11.93^b^	13.34^b^	1.30
B (%)	74.47^ab^	69.72^b^	80.27^a^	77.05^ab^	80.98^a^	1.06
Kd (/h)	0.05^b^	0.09^a^	0.05^b^	0.09^a^	0.11^a^	0.01
ED (6%/h)	43.97^c^	63.85^a^	46.96^c^	58.77^b^	66.25^a^	1.04
**Protein**						
A (%)	76.47^a^	50.70^d^	62.94^c^	62.36^c^	66.42^b^	0.42
B (%)	23.53^d^	44.62^a^	35.92^b^	34.89^b^	30.06^c^	0.45
Kd (/h)	0.03^c^	0.13^a^	0.07^b^	0.13^a^	0.12^ab^	0.00
ED (6%/h)	84.47^b^	80.86^d^	82.13^c^	86.26^a^	86.42^a^	0.19

*^*a,b,ab,c,d,e*^Means in the same row with different superscripts differ (*P* < 0.01). A = the fast degradable fraction; B = the slowly degradable fraction; Kd = degradation rate; A+B = the potential degradable fraction; ED = effective degradability of dry matter, starch and protein; 1/effective degradability in the rumen (assuming rate of passage of 0.06/h^-*1*^); ED = effective degradability of dry matter; 1/effective degradability in the rumen (assuming rate of passage of 0.06/h^-*1*^).*

When heifers were fed the high grain diet, the rapidly degradable DM fraction (A) was highest (*P* < 0.01) for Corn (**Table 3** and **Figure 1B**) and lowest for Fibar. The Kd of DM was fastest (*P* < 0.01) for Xena and lowest for Corn. The potential degradable DM fraction (A+B) (*P* < 0.01) ranked as Corn = Fibar = Hilose = McGwire > Xena, but Xena and McGwire had a higher ED (*P* < 0.01) than Hilose and Corn. The rapidly degradable fraction (A) of starch was highest for Fibar (*P* < 0.01; **Table 3** and **Figure 1D**). Xena had the highest Kd and ED of starch. The ED of protein was ranked as Xena = McGwire > Corn > Hilose > Fibar (**Table 3** and **Figure 1F**).

### Differences in Particle-Associated Bacteria (PAB) Among Corn and the Four Barley Varieties in Heifers Fed a Low Grain Diet

**FIGURE 2:**
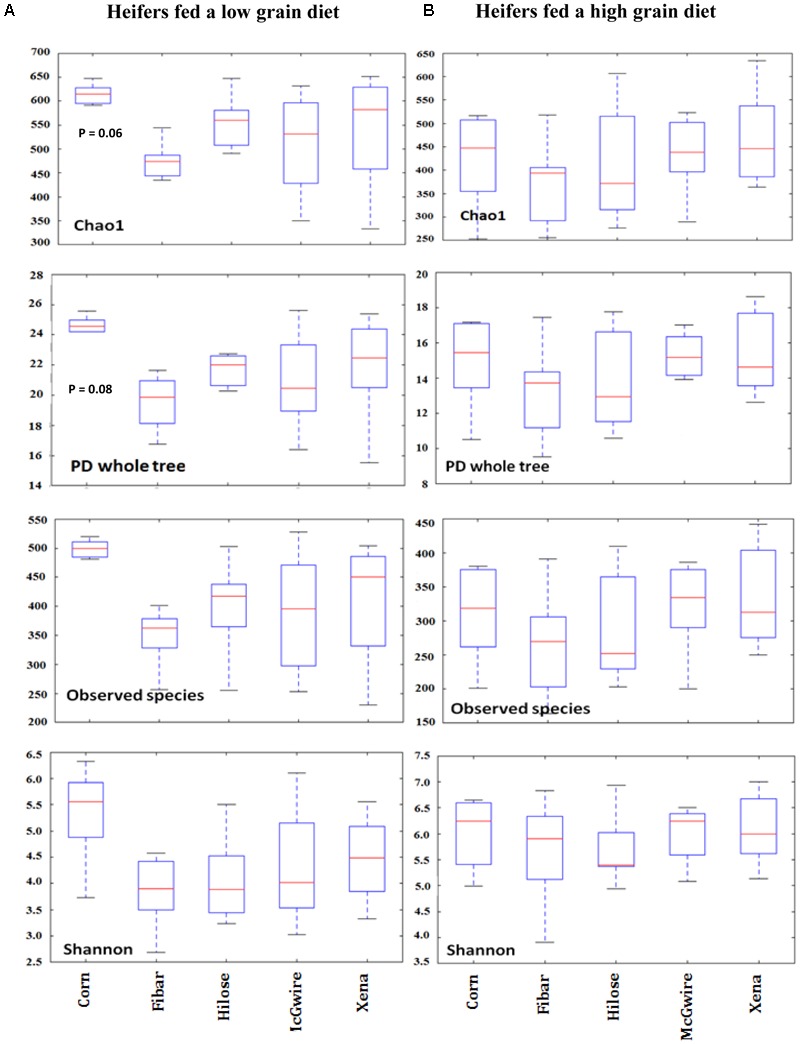
Alpha diversity measures of Chao1, observed species, PD whole tree, and Shannon diversity index associated with biofilms on the surface of corn (*n* = 6) and barley (*n* = 6) incubated in the rumen of heifers fed a low **(A)** or high grain **(B)** diet.

A total of 604,714 raw sequences were generated from the corn and four barley varieties collected from heifers fed the low grain diet. After trimming, quality filtering and chimera removal, a total of 464,120 sequences remained, with an average length of 421 bp. With a cut-off of 97% sequence similarity, 945 bacterial OTUs were obtained. The 945 OTUs were classified into 10 phyla, 29 families, 51 genera, and 1 unclassified group. For grain associated biofilms, species richness, PD, observed species and evenness were assessed using, Chao1, a PD whole tree, observed OTUs, and Shannon’s diversity index, respectively (**Figure 2A**). Good’s coverage illustrated similar sequence coverage among grain samples (Supplementary Figure 1A). We found no difference in observed species or Shannon diversity among corn and barley varieties. However, there was a tendency for Choa1 (*P* = 0.06) and PD (*P* = 0.08) to differ between corn and barley varieties.

**FIGURE 3:**
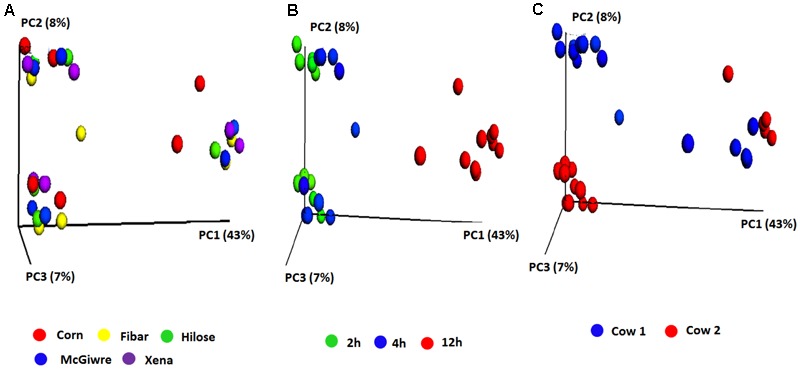
Principle coordinate analysis (PCoA) plots of the unweighted UniFrac distances for biofilm associated bacteria on corn and four barley grain varieties in heifers fed a low grain diet **(A)** corn and four barley varieties, **(B)** sampling time and **(C)** heifer.

Bacterial communities did not differ among barley varieties (**Figure 3A**), but interestingly, the bacterial communities associated with corn and barley grains at 12 h of incubation tended to separate from those at 2 and 4 h (**Figure 3B**). Animal variation was observed at 2 and 4 h. Despite this, the microbiota became less dissimilar by 12 h showing that initial differences by animal were reduced as fermentation progressed (**Figure 3C**).

**FIGURE 4:**
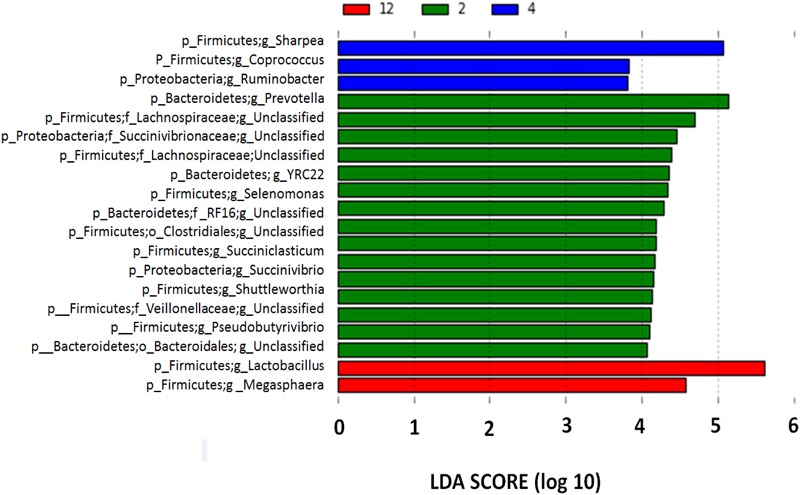
Differentially abundant genera associated with barley grain after 2, 4, and 12 h incubation in the rumen of heifers fed a low grain diet from LEfSe analysis. With a log LDA score above 2.00. Lower case letter before taxonomy indicates phylum (p_), family (f_), or genus (g_).

**FIGURE 5:**
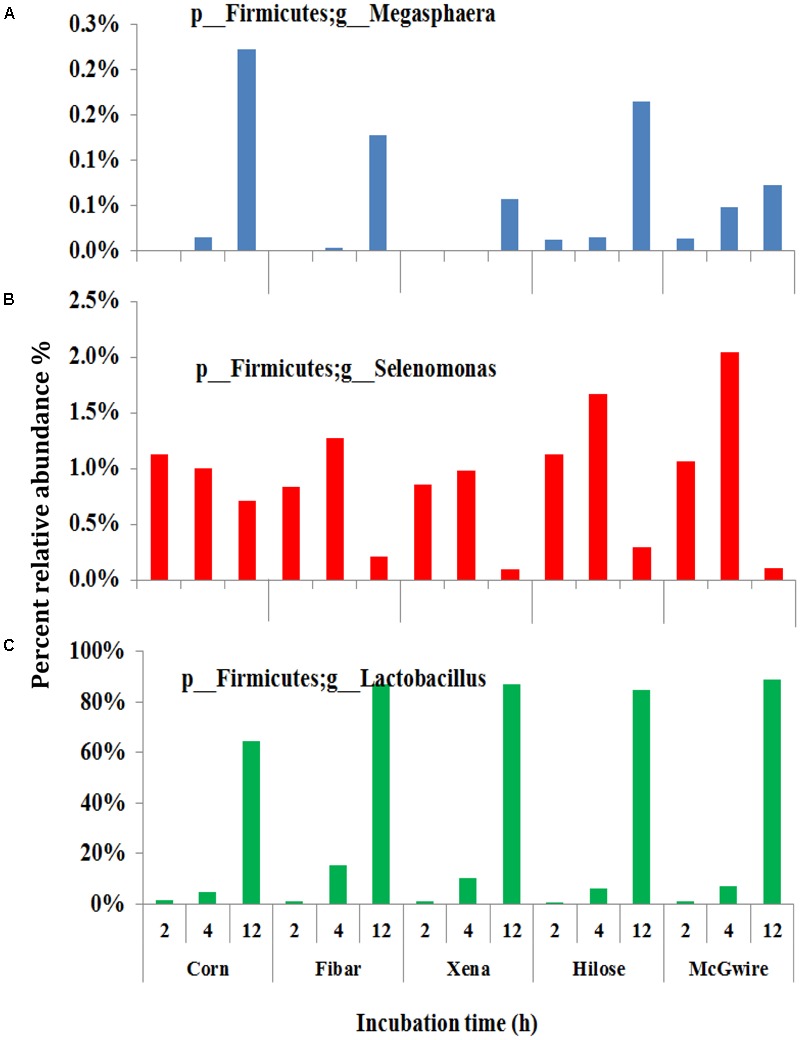
The lactic acid-utilizing bacteria **(A,B)** and *Lactobacillus* **(C)** associated with the biofilms on grains incubated for different times in heifers fed a low grain diet. Lower case letter before taxonomy indicates phylum (p_) or genus (g_).

**FIGURE 6:**
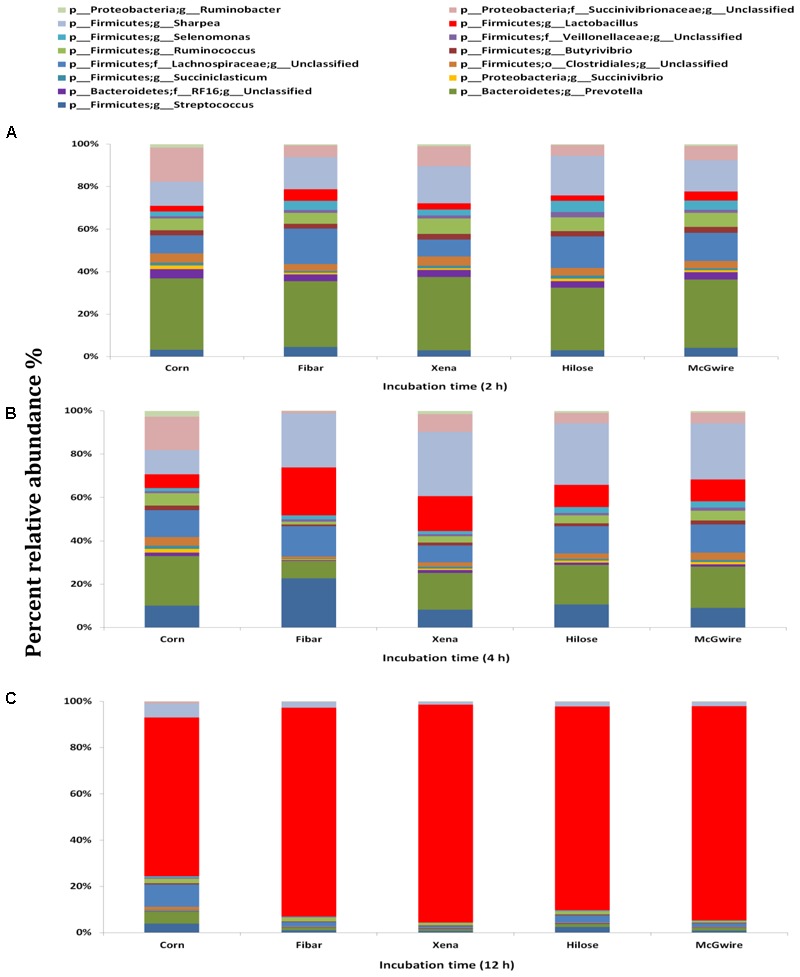
Fifteen most abundant genera within the bacterial biofilm communities associated with corn and four barley varieties incubated in the rumen of heifers fed a low grain diet for **(A)** 2 h, **(B)** 4 h, or **(C)** 12 h. Lower case letter before taxonomy indicates phylum (p_), family (f_), order (o_), or genus (g_).

Although the bacterial communities did not differ among barley varieties, there were noticeable differences in the bacterial communities during the incubation (**Figure 4**). At 2 h, 15 genera were noticeably higher in the biofilm associated with barley grain as compared to biofilms after 4 and 12 h of incubation. After 4 h of incubation, *Sharpea, Coprococcus*, and *Ruminobacter* were higher in biofilms associated with barley grain than after 2 and 12 h. After 12 h of incubation, *Lactobacillus* and *Megasphaera* were more abundant in the biofilm than at 2 and 4 h. In addition, **Figure 5** shows that the increase in lactic acid-utilizing bacteria (*Megasphaera*; **Figure 5A** and *Selenomonas*; **Figure 5B**) correlated with changes in lactic acid producing bacteria (*Lactobacillus*; **Figure 5C**). *Lactobacillus* was positively correlated (*r* = 0.76) with *Megasphaera* and negatively correlated (*r* = -0.80) with *Selenomonas.* This highlights the relationship between lactic acid producers and utilizers and the use of lactate as a substrate in the rumen. *Prevotella* was the most dominant genus accounting for 16.5, 6.1, 10.8, 6.3, and 7.8% in corn, Fibar, Xena, Hilose, and McGwire, respectively, after 2 h of incubation (**Figure 6A**). After 4 h of incubation, *Prevotella* (16.3%) predominanted, followed by unclassified *Succinivibrionaceae*, unclassified *Lachnospiraceae*, and *Sharpea* in corn. In contrast, *Sharpea* was the most abundant in Fibar, Hilose, McGwire, and Xena, representing 17.3, 16.8, 17.9, and 19.1% of the population, respectively (**Figure 6B**). After 12 h of incubation, *Lactobacillus* was the most abundant in both corn and barley (**Figure 6C**).

**FIGURE 7:**
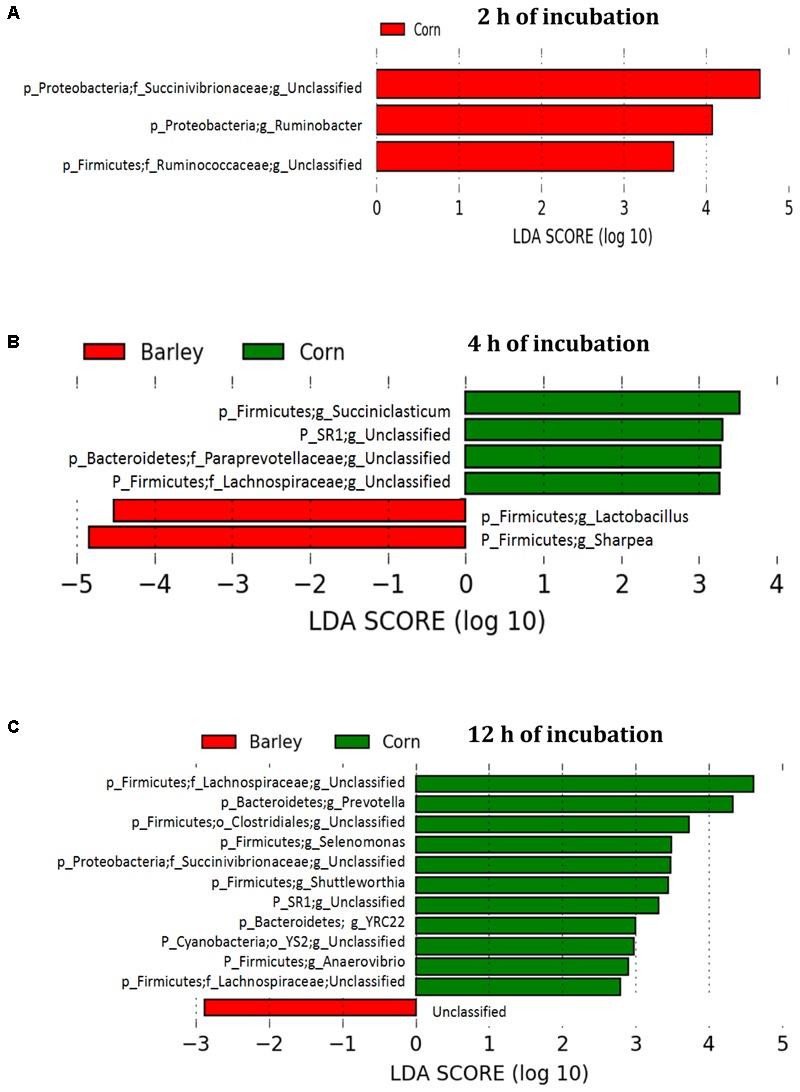
Comparison of corn and barley grain associated biofilms illustrating an increase in the abundance select genera after **(A)** 2 h, **(B)** 4 h, and **(C)** 12 h of incubation in the rumen of heifers fed a low grain diet. LEfSe analysis used a log LDA score above 2.00. Lower case letter before taxonomy indicates phylum (p_), family (f_), order (o_), or genus (g_).

The comparison of corn and barley grain showed that three genera including an unclassified *Succinivibrionaceae, Ruminobacter*, and an unclassified *Ruminococcaceae* significantly increased in corn than barley grain after 2 h of incubation (**Figure 7A**). At this time, four genera (*Succiniclasticum*, unclassified SR1, unclassified *Paraprevotellaceae*, and unclassified *Lachnospiraceae*) were higher (*P* < 0.05) in corn than barley, whereas *Lactobacillus* and *Sharpea* were higher in barley than corn (**Figure 7B**). Moreover, at 12 h incubation, unclassified *Lachnospiraceae, Prevotella*, unclassified *Clostridiales, Selenomonas*, unclassified *Succinivibrionaceae*, unclassified SR1, *YRC22*, unclassified YS2, and *Anaerovibrio* were higher (*P* < 0.05) in corn than in barley varieties (**Figure 7C**).

### Differences in Particle-Associated Bacteria (PAB) Among Corn and the Four Barley Varieties in Heifers Fed a High Grain Diet

With the high grain diet, a total of 395,972 raw sequences were generated. After trimming, quality filtering and chimera checking, a total of 156,814 sequences remained; with an average length of 400 bp. The reads were clustered into 815 OTUs, and their representative sequences were used in taxonomic analysis. The 815 OTUs were classified into 7 phyla, 29 families, 53 genera and 1 unclassified group. Species diversity, richness and evenness were calculated as described above. No significant differences in Chao1 (species richness), observed species (observed OTUs), PD whole tree, or Shannon’s diversity index were observed among corn and the four barley varieties (**Figure 2B**). Good’s coverage was also similar among grain types (Supplementary Figure 1B). Our results showed that the PCoA in heifers fed the high grain diet produced results that were similar to that observed when the heifers were fed a low grain diet. Bacterial communities clustered more by individual heifer at early incubation times (2 and 4 h), but after 12 h biofilms had matured and were similar between animals. In heifers fed the high grain diet, *Prevotella*, unclassified *Succinivibrionaceae*, and unclassified *Ruminococcaceae* were higher (*P* < 0.05) in barley grain after 2 h than after 4 and 12 h of incubation, and *Lactobacillus* was higher (*P* < 0.05) at 12 h than at the other incubation times (data not shown).

**FIGURE 8:**
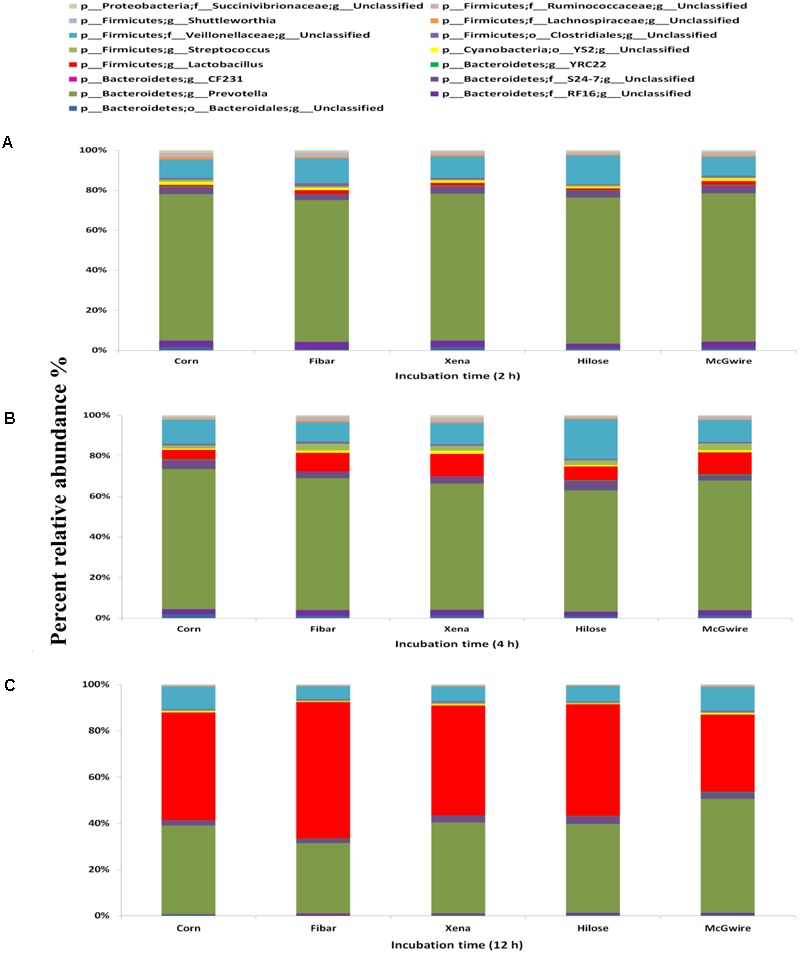
Fifteen most abundant genera within the bacterial biofilm communities associated with corn and four barley varieties incubated in the rumen of heifers fed a high grain diet for **(A)** 2 h, **(B)** 4 h, or **(C)** 12 h. Differing durations of incubation in the rumen of heifers fed a high grain diet. Lower case letter before taxonomy indicates phylum (p_), family (f_), order (o_), or genus (g_).

A total of 53 genera were identified across all samples, with the fifteen most abundant genera displayed in **Figure 8**. After 2 and 4 h of incubation, *Prevotella* was most abundant in corn and barley grains, followed by unclassified *Veillonellaceae* (**Figures 8A,B**). Whereas after 12 h of incubation, *Lactobacillus* was higher in corn (44.5%), Fibar (57.0%), Xena (45.0%), Hilose (45.1%), and McGwire (31.5%) than after 2 or 4 h of incubation (**Figure 8C**). Compared to barley after 2 h of incubation, corn had higher (*P* < 0.05) unclassified *Ruminococcaceae* and *Fibrobacter* than barley grain and after 4 h of incubation, CF231 was higher (*P* < 0.05) in corn than barley (data not shown).

### Bacterial Morphologies Associated With Barley Grain

**FIGURE 9:**
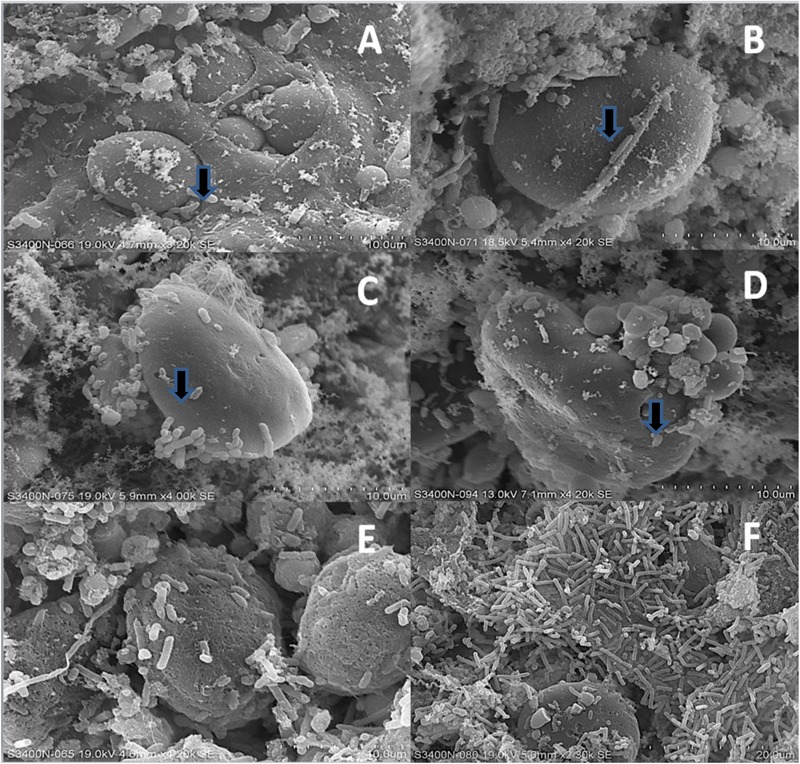
Rumen bacterial biofilms on the surface of grains as viewed using scanning electron microscopy (SEM) after 2, 4, and 12 h of ruminal incubation. **(A)** Fibar at 2 h. **(B)** McGwire at 2 h. **(C)** Fibar at 4 h. **(D)** McGwire at 4 h. **(E)** Fibar at 12 h. **(F)** McGwire at 12 h.

After 2 h of incubation, SEM images showed initial colonization of starch granules by rumen bacteria, but digestive pits on the surface of starch granules were not apparent (arrows, **Figures 9A,B**). By 4 h, the formation of rich biofilms with visible digestive pits on the surface of starch granules was evident (arrows, **Figures 9C,D**). After 12 h of incubation in the rumen, the protein matrix of starch granules was also undergoing invasion by bacteria and penetration of starch granules by starch-digesting bacteria was observed (**Figures 9E,F**).

## Discussion

### *In Situ* Degradability of Different Barley Varieties in Heifers Fed Low or High Grain Diets

Formation of microbial biofilms is a perquisite for efficient ruminal feed digestion (McAllister et al., 1994) as adherent bacteria have been estimated to be responsible for 80% of the endoglucanase activity, 70% of the amylase activity (Minato et al., 1966), and 75% of the protease activity (Brock et al., 1982) in the rumen.

Our *in situ* degradability study showed that the normal barley varieties of McGwire and Xena, which generally contain an amylose:amylopectin ratio of 1:3, exhibited a higher DM digestibility as compared to high amylose (Hilose) and high amylopectin (Fibar) barley or corn (**Table 3**). Similarly, Stevnebø et al. (2009) showed that low amylose barley varieties had a greater effective Kd (0.148/h) compared to normal amylose (0.115/h) and high amylose (0.102/h) varieties. However, studies comparing the impact of the amylose/amylopectin ratio on starch digestibility in the rumen are limited and inconsistent. Philippeau and Michalet-Doreau (1997) concluded that ruminal starch degradability was not related to amylose content in corn starch, as measured by the *in situ* bag technique. Interestingly, in our study, corn had lower *in situ* rumen degradability in both diets as compared to barley. According to McAllister et al. (1993), differences in ruminal starch digestibility between corn and barley grain are related to differences in the composition and structure of the endosperm rather than starch granules. Moreover, other studies showed that the reduced digestibility of starch in corn was attributable to the protein matrix surrounding starch granules (Weurding et al., 2001). In corn, starch granules may be so tightly associated with the protein matrix (Philippeau et al., 2000; McAllister and Riberio, 2013), that starch resists degradation by rumen bacteria (McAllister et al., 1993; Philippeau et al., 2000), accounting for the lower rate of starch digestion in corn as compared to barley.

Even though our study observed differences in the degradability of barley varieties in both diets, we did not identify clear differences in the biofilm bacterial communities associated with different barley varieties in heifers fed either low or high barley grain diets. This suggests that the variation in degradability among barley varieties was a result of differences in the chemical composition of the grains, and not the bacterial composition of the biofilms partaking in fermentation. However, our study showed changes in the microbiota over time (i.e., 2, 4, and 12 h of incubation) in all grains, regardless of diet. Interestingly, biofilms exhibited different bacterial diversity between corn and barley irrespective of time and diet. This is probably a result of structural differences in the protein matrix of the grain (i.e., corn versus barley) affecting surface biofilm development rather than differences in starch composition (i.e., among barley varieties). However, the bacterial communities were higher in bacterial diversity and complexity in heifers fed high forage than in those fed high grain. Generally, biofilms are formed by dominant bacteria and evolve with incubation time, possibly in relation to changes in the composition of the feed as digestion proceeds.

### Temporal Microbial Colonization of Corn and Barley Grain Within the Rumen

The biofilms responsible for digestion of feed particles develop overtime, with three successive populations contributing to the biofilm (McAllister et al., 1994; Huws et al., 2016). The first population consists of those loosely associated bacteria that utilize water-soluble sugars and proteins that are available in the early stages of digestion. This population is followed by more tightly associated populations that adhere to the surface of plant cell walls and starch granules, contributing to the establishment of a stable biofilm. Finally, a third population integrates into the biofilm, utilizing the fermentation end products produced by the tightly associated population. Huws et al. (2013) showed that the primary colonizing bacteria were associated with fresh perennial rye grass within 1–2 h after its introduction into the rumen. Secondary colonizers were shown to join the biofilm of fresh perennial ryegrass within 4–8 h. Interestingly, using denaturing gradient gel electrophoresis, rumen biofilms associated with Chinese wild rye were shown to continue to evolve even after 6 and 12 h of incubation in the rumen (Sun et al., 2008). These studies support our microscopy data (**Figure 9**), which showed that at 2 h incubation, bacteria initiated colonization of starch granules, but mature biofilms had not formed and bacterial invasion of starch granules and the protein matrix had not yet occurred. At 4 h of incubation, bacterial penetration of starch granules was apparent, and rich biofilms were visible on the surface of both starch granules and the protein matrix after 12 h of incubation.

### Temporal Microbial Colonization Among Corn and the Four Barley Varieties in Heifers Fed Low Grain Diet

Even though the bacterial communities in biofilms associated with the four barley varieties did not differ, the bacterial composition of the biofilms did dramatically change over time (**Figure 4**). The diversity of the primary colonizers was greater than that observed as the biofilm advanced toward maturity. This may reflect the rich variety of soluble and insoluble nutrients available for digestion during the early stages of biofilm formation. As digestion proceeds, soluble nutrients are exhausted and the biofilm becomes more mature and less compositionally diverse (Watnick and Koltr, 2000). Similarly, Huws et al. (2016) found a higher bacterial diversity associated with fresh perennial ryegrass after 1–2 h of incubation in the rumen as compared to 4–6 h of incubation. Compared to barley, primary biofilm formation in corn had higher unclassified *Succinivibrionaceae, Ruminobacter*, and unclassified *Ruminococcaceae*, a response that may reflect the higher level of starch in corn. Huws et al. (2016) proposed that primary colonizing bacteria associate first with soluble nutrients, with secondary colonizers being more adept at digesting starch and plant cell walls. During secondary colonization of barley grain, *Sharpea, Coprococcus*, and *Ruminobacter* increased, all of which have been associated with starch digestion. As soluble carbohydrates are utilized, the digestion of starch likely intensifies. Digestion is likely enhanced as primary colonizers degrade physical barriers and increase the access of secondary colonizers to starch. Comparison of the secondary colonizers found that four genera (*Succiniclasticum*, unclassified SR1, unclassified *Paraprevotellaceae*, and unclassified *Lachnospiraceae*) were more abundant in biofilms associated with corn than barley. Whereas at this same time, *Lactobacillus*, and *Sharpea* were more abundant in biofilms associated with barley than corn. The probable explanation is that corn and barley grain differ in starch structure in relation to interaction with the protein matrix. The starch endosperm of corn is tightly associated with the protein matrix, which resists degradation by ruminal bacteria (Philippeau et al., 2000). Whereas, the starch of barley grain is loosely associated with the protein matrix, easily exposed and digested by ruminal bacteria.

In general, tertiary biofilms became less diverse with increasing time in the rumen. This study showed that *Lactobacillus* and *Megasphaera* increased in the tertiary biofilm associated with barley grain during primary and secondary colonization (**Figure 4**). Generally, *Lactobacillus* is most often associated with ruminal acidosis (Sato, 2016) due to its capacity to rapidly produce large amounts of lactic acid (Hernandez et al., 2007). However, surprisingly, we observed that *Lactobacillus* increased and accounted for a significant proportion of the adherent microbiota of grains, even when heifers were fed low grain diets. We also found that lactic acid-utilizing *Megasphaera* increased within the tertiary biofilm. *Megasphaera* play an important role in preventing the accumulation of lactic acid in the rumen (Castillo-González et al., 2014). Thus, the increase in lactic acid production by *Lactobacillus* is likely offset by an increase in lactic acid utilization by *Megasphaera*. This suggests that *Lactobacillus* is active in starch digestion, even when acidosis is not evident. Counotte et al. (1981) also suggested that *Megasphaera elsdenii* is the main species responsible for lactic acid metabolism and has an important role in the prevention of lactic acidosis.

Unclassified *Lachnospiraceae, Prevotella*, unclassified *Clostridiales*, unclassified S*uccinivibrionaceae, Shuttleworthia*, unclassified SR1, *YRC22*, unclassified YS2, and *Anaerovibrio* were more dominant in tertiary biofilms associated with corn than with barley (**Figure 7C**). This suggests that the nature of the protein matrix or other chemical constituents such as lipid may result in greater bacterial diversity in mature biofilms in corn than in barley. This possibility is also supported by the fact that, *Prevotella* was higher in corn than barley as the superior proteolytic capacity of this bacterium has been well documented (Wallace et al., 1995, 1997). The reason for the increased abundance of unclassified *Clostridiales* during the tertiary biofilm in corn is unclear, but these genera could also be associated with the digestion of protein matrix in corn. Poppi and Quigley (2003) also found an abundance of unclassified *Clostridiales* associated with the higher protein levels in a cottonseed meal supplement. The identification of *Anaerovibrio* a lipolytic bacterium in corn biofilms may also be related to the higher lipid content of this grain.

### Temporal Microbial Colonization Among Corn and the Four Barley Varieties in Heifers Fed High Grain Diet

Biofilm bacterial communities associated with corn and barley grain were also influenced by the level of grain that was fed to heifers. The three genera that were higher during the initial colonization of barley grain included *Prevotella*, unclassified *Succinivibrionaceae*, and unclassified *Ruminococcaceae*. Interestingly, our data showed that *Prevotella* was the most dominant genus in both grain types (i.e., corn vs. barley grain) and diets (i.e., high fiber and high grain diets) during the initiation of biofilm formation. This is probably because of the diversity of species within *Prevotella* and the wide range of metabolic functions they possess. Koike et al. (2003) also showed that *Prevotella* were found to be predominant in the total 16S rRNA sequences retrieved from the particle-associated rumen bacterial communities. It has also been shown that members of this genus participate in both fiber and grain digestion (Bekele et al., 2010) and possess both amylolytic and proteolytic activities (Accetto and Avguštin, 2015; Kishi et al., 2015). As with the low grain diet, lactobacilli were also a predominant member of the mature biofilm colonizing grains in heifers fed the high grain diet.

## Conclusion

Our study showed that standard barley varieties exhibited higher ruminal DM, starch, and protein digestibility than high amylose and amylopectin varieties. However, these differences in digestion were not reflected in the composition of the biofilm communities responsible for the digestion of these grains. *Prevotella* were the predominant primary colonizers of grains in heifers fed either low or high grain diets. Starch digestion was accelerated upon establishment of the secondary colonizers with *Lactobacillus* predominating in mature biofilms that were less diverse than during initial colonization. The inclusion of lactic acid utilizing bacteria within mature biofilms likely plays a significant role in preventing the accumulation of lactic acid in the rumen.

## Author Contributions

HY, JM, and TM designed the study and wrote the manuscript. HY and CZ carried out the experimental procedures and analysis. HY and TM conducted the statistical and bioinformatics analysis. TM provided funding. All the authors reviewed and approved the final manuscript.

## Conflict of Interest Statement

The authors declare that the research was conducted in the absence of any commercial or financial relationships that could be construed as a potential conflict of interest.
