# Supplementary material for: Lactobacilli Are Prominent Members of the Microbiota Involved in the Ruminal Digestion of Barley and Corn
Source: Front Microbiol. 2018 Apr 10;9:718. doi: 10.3389/fmicb.2018.00718 (PMC5902705; doi:10.3389/fmicb.2018.00718)
Supplement: Supplementary file 1 [file Image_1.PDF]

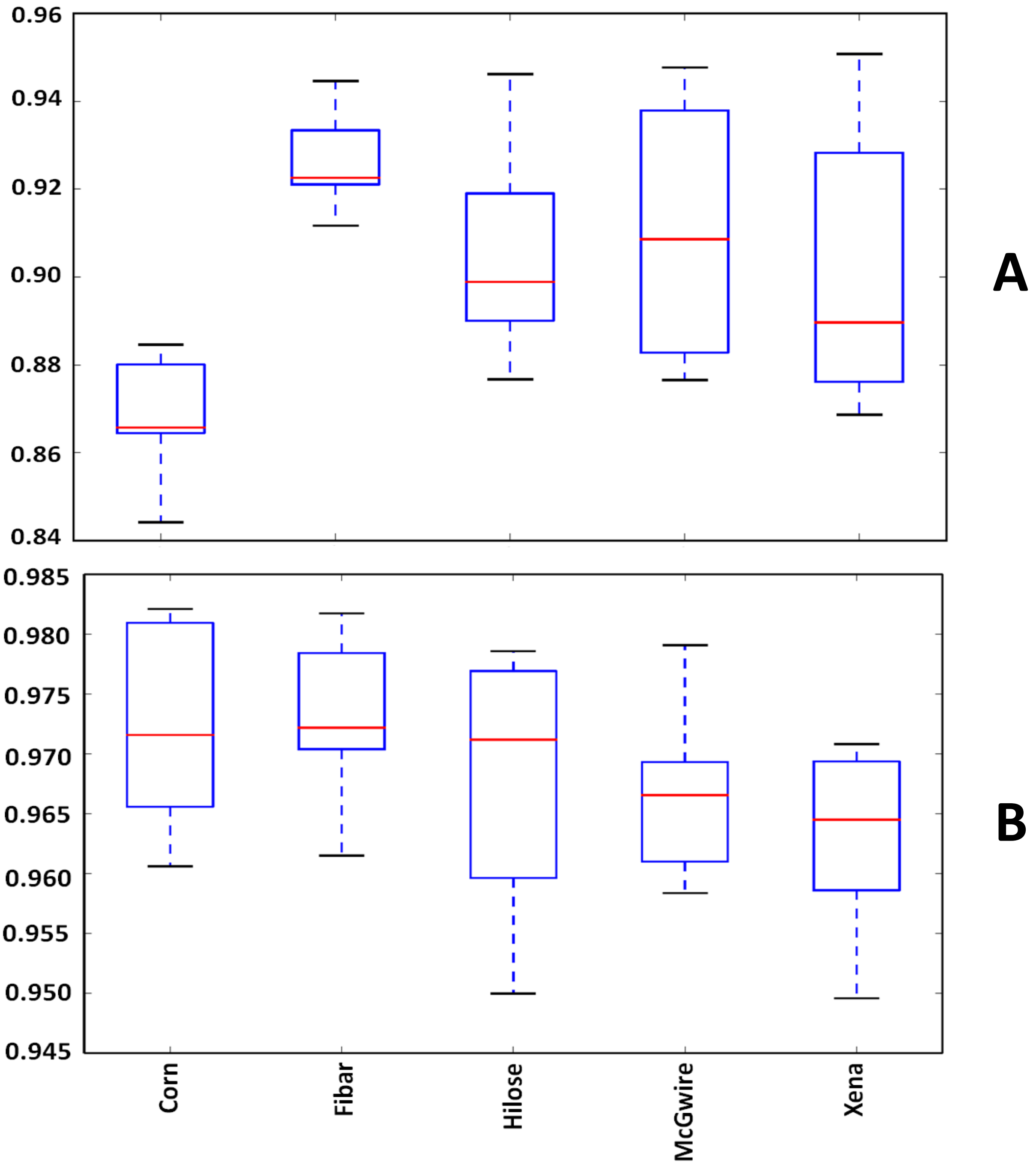

Supplementary Figure 1. Alpha diversity measures of Good's coverage diversity index associated with biofilms on the surface of corn (n=6) and barley (Fibar, Hilose, McGwire and Xena) (n=6) incubated the rumen of heifers fed a low (A) or high grain (B) diet.
